# How Computer Literacy and Socioeconomic Status Affect Attitudes Toward a Web-Based Cohort: Results From the NutriNet-Santé Study

**DOI:** 10.2196/jmir.3813

**Published:** 2015-02-02

**Authors:** Camille Pouchieu, Caroline Méjean, Valentina A Andreeva, Emmanuelle Kesse-Guyot, Philippine Fassier, Pilar Galan, Serge Hercberg, Mathilde Touvier

**Affiliations:** ^1^Sorbonne Paris Cité, Epidemiology and Biostatistics Research Center, Nutritional Epidemiology Research Team (EREN), Inserm U1153; Inra U1125; Cnam; Paris 13, 7 and 5 UniversitiesBobigny cedexFrance; ^2^Public Health Department, Avicenne HospitalBobignyFrance

**Keywords:** computer literacy, Internet, cohort study, attitudes

## Abstract

**Background:**

In spite of the growing literature in the field of e-epidemiology, clear evidence about computer literacy or attitudes toward respondent burden among e-cohort participants is largely lacking.

**Objective:**

We assessed the computer and Internet skills of participants in the NutriNet-Santé Web-based cohort. We then explored attitudes toward the study demands/respondent burden according to levels of computer literacy and sociodemographic status.

**Methods:**

Self-reported data from 43,028 e-cohort participants were collected in 2013 via a Web-based questionnaire. We employed unconditional logistic and linear regression analyses.

**Results:**

Approximately one-quarter of participants (23.79%, 10,235/43,028) reported being inexperienced in terms of computer use. Regarding attitudes toward participant burden, women tended to be more favorable (eg, “The overall website use is easy”) than were men (OR 0.65, 95% CI 0.59-0.71, *P*<.001), whereas better educated participants (>12 years of schooling) were less likely to accept the demands associated with participation (eg, “I receive questionnaires too often”) compared to their less educated counterparts (OR 1.62, 95% CI 1.48-1.76, *P*<.001).

**Conclusions:**

A substantial proportion of participants had low computer/Internet skills, suggesting that this does not represent a barrier to participation in Web-based cohorts. Our study also suggests that several subgroups of participants with lower computer skills (eg, women or those with lower educational level) might more readily accept the demands associated with participation in the Web cohort. These findings can help guide future Web-based research strategies.

## Introduction

The use of Web-based questionnaires in prospective epidemiological studies has increased steadily over the past decade [1-11], driven by substantial logistic simplification (cost-effectiveness, convenience regarding place/time of survey completion, ease of converting data to an analyzable format) and scientific advantages (improved quality and quantity of exposure measurement, complex research designs, study of sensitive topics or rare conditions) of Web compared to traditional methods (paper-and-pencil questionnaires, face-to-face interviews). Other features, such as recruitment of very large samples and hard-to-reach populations (low socioeconomic strata, risky behavior profiles), quick returns, and data management facility and flexibility, are also strengths of e-epidemiology [12,13]. This medium for data collection is being increasingly favored given the growth of Web access and the use of personal computers [2]. In the United States, 75% of households were connected to the Internet in 2012 [14]. In France, this proportion was similar in 2012 (78%) compared to 54% in 2007 [15]. In this context, it remains unknown the extent to which low computer and Internet literacy represent a barrier to participation in Web-based studies. Very few Web-based epidemiological studies have provided information about the level of computer and Internet skills of their participants [16].

Another key question pertains to perceived respondent burden in Web-based studies (eg, regarding frequency and length of questionnaires) and its variability according to computer skills, age, gender, and the educational level of participants. Indeed, it has been shown that perceived ease and user-friendliness of the study website and the level of perceived difficulty of questionnaire completion may have a major impact on response and completion rates in e-epidemiology [17-19]. However, data are lacking in the literature regarding opinions and acceptance of respondent burden in Web-based studies and its correlation with computer skills of participants. Filling this knowledge gap would be useful for ongoing and future Web-based cohort studies, for instance, for improving and adapting the design of questionnaires according to the target population.

Thus, our objectives were (1) to assess computer and Internet skills of participants in a large Web-based cohort (the NutriNet-Santé study) and (2) to compare their attitude toward study demands according to sociodemographic background and computer literacy.

## Methods

### Participants

The ongoing NutriNet-Santé study is the first large-scale Web cohort set up to investigate the relationships between nutrition and health in the general population [4]. It was launched in France in May 2009 to evaluate the determinants and characteristics of eating behavior and the relationship between nutrition and chronic disease risk. Participants are recruited by a vast multimedia campaign. Inclusion criteria are age ≥18 years and access to the Internet. Registration and participation take place online using a dedicated and secure website. Participants receive regular emails informing them about a new questionnaire available for completion and communicating study results and newsletters. The study website also provides general information on health and nutrition topics and on scientific publications related to the cohort. Contact between investigators and study participants is established by the Internet (dedicated website and emails). This study was approved by the Institutional Review Board of the French Institute for Health and Medical Research (IRB Inserm No 0000388FWA00005831) and the “Comission Nationale de l’Informatique et des Libertés” (CNIL No 908450 and No 909216).

### Data Collection

Participants completed a baseline set of 5 self-administered, Web-based questionnaires on sociodemographic and lifestyle characteristics, anthropometrics, dietary intake (using repeated 24-hour dietary records), and physical activity along with health status. Thereafter, these baseline questionnaires are administered each year to update the information. All these instruments have been tested against traditional assessment methods (paper questionnaires or interview by a health professional) [20-22].

Data on sociodemographic characteristics included age, gender, education, and occupation. In October 2013, participants were sent a computer literacy Web questionnaire in which they were asked to self-evaluate their level of computer skills (novice, inexperienced, experienced, or expert) and to report if they were able to perform specific computer/Internet tasks in order to evaluate their computer literacy profile. This questionnaire also aimed to gather information on perceived respondent burden in the NutriNet-Santé study (ease of website use, interest in the information section, acceptable frequency and completion time of the questionnaires, satisfaction with the communication between study staff and participants, potential interest in a NutriNet-Santé smartphone application). This questionnaire was not mandatory and no reminders were sent to nonresponders.

### Statistical Analyses

From the 123,984 participants included in the NutriNet-Santé study between May 2009 and October 2013, 43,028 individuals (34.70%) returned the optional questionnaire on computer skills and Internet use.

Sociodemographic characteristics are presented in a frequency/percent format for the entire sample: gender, age (<30 years, 30-44 years, 45-59 years, ≥60 years), educational level (>12 or ≤12 years of schooling), and occupational category (farmers, manual workers, employees, intermediate professions/ skilled office work, self-employed, managerial staff, never employed). Similarly, overall and task-specific computer skills are presented in a frequency/percent format.

Opinions about respondent burden in the NutriNet-Santé study were compared by multivariate unconditional logistic regression analyses according to sociodemographic characteristics (gender: women vs men; age: >50 years vs ≤50 years; education: >12 years vs ≤12 years of schooling) and self-evaluated level of computer skills (experienced-expert vs novice-inexperienced). Actual and self-perceived acceptable questionnaire completion times were compared by multivariate linear regression analyses according to the same sociodemographic characteristics and self-evaluated level of computer skills. These variables were log-transformed to improve normality. Adjusted means and standard errors (SE) were reported. Multivariate models were mutually adjusted for gender, age, educational level, and self-evaluated level of computer skills.

A *P* value <.05 was considered statistically significant. Analyses were carried out with SAS version 9.3 (SAS Institute Inc, Cary, NC, USA).

## Results

Sociodemographic characteristics of the study population (N=43,028) are presented in Table 1. In all, 76.07% (32,731/43,028) of the participants were women and the mean age was 51.2 years (SD 14.5). The sample included 35.16% (15,130/43,028) managerial staff, 28.37% (12,209/43,028) intermediate professions/skilled office work, 26.83% (11,544/43,028) technical/routine occupations, 2.83% (1217/43,028) self-employed, 2.77% (1191/43,028) farmers and manual workers, and 4.04% (1737/43,028) never-employed participants (current occupation or most recent job for retired or currently unemployed participants).

**Table 1:** Baseline characteristics of respondents to the computer literacy questionnaire, NutriNet-Santé cohort, France, 2013.

Individual characteristics		French population,^a^ % (N=48,730,086)	Full NutriNet-Santé cohort, n (%) (N=123,984)	Respondents to the computer literacy questionnaire, n (%) (N=43,028)
**Gender**				
	Female	52.4	96,912 (78.16)	32,731 (76.07)
	Male	47.6	27,072 (21.84)	10,297 (23.93)
**Age categories (years)**				
	<30	22.8	18,518 (14.94)	4298 (9.99)
	30-44	24.8	40,432 (32.61)	10,626 (24.70)
	45-59	24.8	35,923 (28.97)	13,623 (31.66)
	≥60	27.6	29,111 (23.48)	14,481 (33.65)
**Educational level**				
	Advanced/graduate degree (≥17 y of schooling)	13.0	40,274 (32.48)	14,457 (33.60)
	Undergraduate degree (13-16 y of schooling)	11.9	36,579 (29.50)	12,663 (29.43)
	Secondary degree (≤12 y of schooling)	17.6	43,070 (34.74)	14,526 (33.76)
	Elementary degree (≤5 y of schooling)	40.2	2375 (1.92)	942 (2.19)
	No degree	17.3	1686 (1.36)	440 (1.02)
**Level of computer skills**				
	Expert			5365 (12.47)
	Experienced			27,428 (63.74)
	Novice			9288 (21.59)
	Inexperienced			947 (2.20)

^a^ National Institute of Statistics and Economic Studies (INSEE), 2014.

### Computer and Internet Skills

A substantial proportion of the participants (23.79%, 10,235/43,028) evaluated themselves as novice or inexperienced in computer use. This was illustrated by the description of specific skills (Table 2). For instance, 36.42% (14,881/43,028) did not know usual keyboard shortcuts (eg, CTRL+C, CTRL+V), 38.74% (16,667/43,028) did not know how to post messages on discussion forums, and 37.53% (16,147/43,028) did not know how to place a telephone call by the Internet.

**Table 2:** Self-reported computer and Internet skills of participants (n=43,028), NutriNet-Santé Study, France, 2013.

Self-reported computer and Internet skills		Positive responses, n (%)
**Computer skills**		
	Copy or move a file or folder	39,693 (92.25)
	Transfer files between a computer and a device (digital camera, USB stick, cell phone, etc)	37,827 (87.91)
	Burn or copy a CD/DVD	32,466 (75.45)
	Install new devices (modem, printer, scanner, webcam, etc)	31,465 (73.13)
	Use basic arithmetic formulas in a spreadsheet (Excel, Open Office Calc, etc)	27,357 (63.58)
	Create electronic presentation	25,102 (58.34)
	Use keyboard shortcuts (CTRL+C, CTRL+V, CTRL+X, etc)	28,147 (65.42)
	Compress/decompress (or zip) files	23,794 (55.30)
	Install or update an operating system (Windows XP, Windows 7, Windows 8, Mac OS, Linux)	18,992 (44.14)
	Change or check the configuration settings for a software	18,352 (42.65)
	Upgrade a computer (desktop or laptop) by changing the hard disk or memory (RAM)	6614 (15.37)
	Write a computer program (C, C++, PHP, HTML, Java, etc)	3359 (7.81)
**Internet skills**		
	Use a search engine (Google, Yahoo, Bing, etc)	42,288 (98.28)
	Send emails with attached files (document, photo, etc)	41,333 (96.06)
	Fill in administrative forms online	39,718 (92.31)
	Buy or sell goods and services online	36,509 (84.85)
	Use instant messaging software (Yahoo, Facebook, Skype, Windows Messenger, Google Talk, etc)	29,522 (68.61)
	Phone by connecting to the Internet (Skype, Yahoo Messenger, Google Talk, etc)	26,881 (62.47)
	Post messages in online discussion forum or chat site	26,361 (61.26)
	Download movies, music, games, etc	26,019 (60.47)
	Change the security parameters of a Web browser	24,019 (55.82)
	Upload text, games, photos, movies or music (on social networks like Facebook or Twitter, for example)	21,025 (48.86)
	Create and manage a blog	10,158 (23.61)
	Create and manage a website	6435 (14.96)

### Opinions and Attitudes Toward Study Demands

Overall acceptance of the study was high: 94.50% (40,662/43,028) reported that the website use was easy, 91.32% (39,293/43,028) were satisfied with the current frequency of questionnaire administration, and 25.22% (10,852/43,028) reported that even a higher frequency than the one currently employed (ie, 1 questionnaire/month) would be acceptable (Tables 3 and 4).

**Table 3:** Opinions and attitudes toward the NutriNet-Santé Study demands according to gender and age, NutriNet-Santé Study, France, 2013.^a^

Opinions and attitudes		n (%)	Gender (female vs male)		Age (>50 y vs ≤50 y)	
			OR (95% CI)	*P* ^b^	OR (95% CI)	*P* ^b^
**The overall website use is easy**				<.001		.06
	Agree	40,662 (94.50)	1.00 (Reference)		1.00 (Reference)	
	Disagree	2366 (5.50)	0.65 (0.59, 0.71)		1.09 (1.00, 1.19)	
**The information section is interesting**				<.001		<.001
	Agree	29,231 (67.93)	1.00 (Reference)		1.00 (Reference)	
	Disagree	2042 (4.75)	0.61 (0.55, 0.68)		0.62 (0.56, 0.68)	
	I don’t read these sections	11,755 (27.32)	0.90 (0.86, 0.95)		0.52 (0.50, 0.55)	
**What do you think about the frequency of the questionnaire mailing?**				<.001		<.001
	The current frequency suits me	39,293 (91.32)	1.00 (Reference)		1.00 (Reference)	
	I receive questionnaires too often	2900 (6.74)	0.80 (0.73, 0.87)		1.25 (1.15, 1.35)	
	I would like to receive questionnaires more often	835 (1.94)	1.01 (0.85, 1.21)		0.30 (0.25, 0.35)	
**What is the maximum acceptable frequency to complete a questionnaire?**				.07		<.001
	Once per week	2126 (4.94)	0.96 (0.86, 1.07)		0.32 (0.29, 0.36)	
	Once every 2 weeks	8726 (20.28)	0.99 (0.94, 1.06)		0.51 (0.49, 0.54)	
	Once per month (ie, current frequency)	24,936 (57.95)	1.00 (Reference)		1.00 (Reference)	
	Once every 3 months	6138 (14.27)	1.00 (0.94, 1.07)		1.37 (1.29, 1.46)	
	Once every 6 months	829 (1.93)	0.75 (0.64, 0.88)		1.53 (1.31, 1.80)	
	Once per year	273 (0.63)	0.67 (0.52, 0.87)		1.85 (1.39, 2.47)	
**Would you prefer to be contacted by means other than the Internet?**						
	Yes, by mail	2206 (5.13)	1.35 (1.20, 1.52)	<.001	0.57 (0.52, 0.62)	<.001
	Yes, by phone	1123 (2.61)	0.83 (0.73, 0.96)	.01	0.78 (0.69, 0.89)	<.001
	Yes, with a personal appointment	1441 (3.35)	0.77 (0.69, 0.87)	<.001	1.08 (0.96, 1.21)	.20
	Yes, at a meeting	1037 (2.41)	1.31 (1.12, 1.53)	<.001	1.89 (1.64, 2.17)	<.001
	No, the current method suits me	37,221 (86.50)	0.96 (0.89, 1.02)	.20	1.16 (1.10, 1.24)	<.001
**Are you interested in the development of a NutriNet smartphone application?**				<.001		<.001
	Yes	8970 (20.85)	1.00 (Reference)		1.00 (Reference)	
	No	27,385 (63.64)	1.25 (1.18, 1.33)		3.24 (3.07, 3.42)	
	I don’t know	6673 (15.51)	1.10 (1.02, 1.19)		2.28 (2.13, 2.44)	

^a^ Logistic regression analyses mutually adjusted for gender, age, education, and level of computer skills.

^b^ Tests for linear trend were performed using the ordinal score for each category. *P* values for trend are reported.

**Table 4:** Opinions and attitudes toward the NutriNet-Santé Study demands according to education and self-evaluated level of computer skills, NutriNet-Santé Study, France, 2013.^a^

Opinions and attitudes		n (%)	Educational level (>12 y vs ≤12 y of schooling)		Level of computer skills (experienced vs novice)	
			OR (95%CI)	*P* ^b^	OR (95%CI)	*P* ^b^
**The overall website use is easy**				<.001		<.001
	Agree	40,662 (94.50)	1.00 (Reference)		1.00 (Reference)	
	Disagree	2366 (5.50)	1.45 (1.32, 1.59)		0.82 (0.74, 0.91)	
**The information section is interesting**				<.001		<.001
	Agree	29,231 (67.93)	1.00 (Reference)		1.00 (Reference)	
	Disagree	2042 (4.75)	2.05 (1.85, 2.28)		1.16 (1.03, 1.31)	
	I don’t read these sections	11,755 (27.32)	2.17 (2.07, 2.28)		1.14 (1.07, 1.20)	
**What do you think about the frequency of the questionnaire mailing?**				<.001		.10
	The current frequency suits me	39,293 (91.32)	1.00 (Reference)		1.00 (Reference)	
	I receive questionnaires too often	2900 (6.74)	1.62 (1.48, 1.76)		0.95 (0.86, 1.04)	
	I would like to receive questionnaires more often	835 (1.94)	0.61 (0.53, 0.70)		1.18 (0.98, 1.42)	
**What is the maximum acceptable frequency to complete a questionnaire?**				<.001		<.001
	Once per week	2126 (4.94)	0.68 (0.62, 0.74)		1.14 (1.01, 1.28)	
	Once every 2 weeks	8726 (20.28)	0.98 (0.93, 1.04)		1.18 (1.11, 1.26)	
	Once per month (ie, current frequency)	24,936 (57.95)	1.00 (Reference)		1.00 (Reference)	
	Once every 3 months	6138 (14.27)	1.03 (0.97, 1.09)		0.95 (0.89, 1.01)	
	Once every 6 months	829 (1.93)	1.15 (0.99, 1.33)		0.83 (0.71, 0.98)	
	Once per year	273 (0.63)	1.04 (0.82, 1.35)		0.87 (0.66, 1.15)	
**Would you prefer to be contacted by means other than the Internet?**						
	Yes, by mail	2206 (5.13)	0.70 (0.64, 0.76)	<.001	0.50 (0.46, 0.55)	<.001
	Yes, by phone	1123 (2.61)	0.85 (0.75, 0.96)	.01	0.67 (0.59, 0.77)	<.001
	Yes, with a personal appointment	1441 (3.35)	0.97 (0.86, 1.08)	.50	0.71 (0.63, 0.80)	<.001
	Yes, at a meeting	1037 (2.41)	0.87 (0.77, 0.99)	.04	0.97 (0.84, 1.12)	.70
	No, the current method suits me	37,221 (86.50)	1.25 (1.18, 1.33)	<.001	1.62 (1.52, 1.72)	<.001
**Are you interested in the development of a NutriNet smartphone application?**				.001		<.001
	Yes	8970 (20.85)	1.00 (Reference)		1.00 (Reference)	
	No	27,385 (63.64)	1.00 (0.95, 1.06)		0.40 (0.38, 0.43)	
	I don’t know	6673 (15.51)	0.90 (0.84, 0.97)		0.52 (0.48, 0.57)	

^a^ Logistic regression analyses mutually adjusted for gender, age, education and level of computer skills.

^b^ Tests for linear trend were performed using the ordinal score for each category. *P* values for trend are reported.

In all, 86.50% (37,221/43,028) of the respondents preferred the current communication modes (email and Internet website) and did not wish to be contacted by any other means (telephone, postal mail, or face-to-face interaction).

Despite their relatively lower computer skills (*P*<.001), women were more positive than men regarding facility of use of the study website (“The overall website use is easy” OR 0.65, 95% CI 0.59-0.71, *P*<.001), reported more interest in the information section (*P*<.001) and supported the current frequency of questionnaire administration (*P*<.001) (Table 3). Alternative contact modes (eg, postal mail or participant in-person meetings) were more frequently endorsed by women, whereas other communication modalities (eg, telephone or direct appointments with study staff) were more likely to be endorsed by men.

Older participants (>50 years) were more interested in the information section on the website (*P*<.001, Table 3) yet also more inclined to report dissatisfaction with the number of administered questionnaires (“I receive questionnaires too often” ) compared with participants aged 50 years or younger (OR 1.25, 95% CI 1.15-1.35, *P*<.001).

Despite having higher computer skills (*P*<.001), participants with higher educational levels appeared to be more demanding regarding the study format compared with participants with ≤12 years of schooling: they were more likely to judge unfavorably the ease of use of the website (OR 1.45, 95% CI 1.32-1.59, *P*<.001) and reported lower interest in the information section as well as dissatisfaction with the number of administered questionnaires (*P*<.001 for all, Table 4). In contrast, the Internet communication mode was preferred over more direct contacts by participants with higher education (*P*<.001).

Participants with higher computer skills were more likely to feel comfortable with the website and with online communication regarding their study participation (both *P*<.001, Table 4).

Overall, 20.85% (8970/43,028) of participants reported being interested in the development of a NutriNet-Santé smartphone app. Men, younger people, and participants with higher self-reported computer skills were more interested in such an app (all *P*<.001).

### Actual and Self-Perceived Acceptable Questionnaire Completion Time

Questionnaire completion time was higher for women, older participants, those with lower educational levels, and lower computer skills (Tables 5 and 6). These categories of participants were more disposed to spend time answering questionnaires, with higher acceptable completion durations declared (*P*=.004 for gender and *P*<.001 for age, educational level, and computer skills).

**Table 5:** Comparison of mean response time for questionnaires (in minutes) according to gender and age, NutriNet-Santé Study, France, 2013.^a^

Type of questionnaire	Gender					Age				
	Female		Male		*P*	>50 y		≤50 y		*P*
	Mean	SEM	Mean	SEM		Mean	SEM	Mean	SEM	
Sociodemographic	14.0	0.05	13.3	0.09	<.001	14.9	0.06	12.4	0.08	<.001
Anthropometric	10.9	0.05	10.4	0.08	<.001	11.6	0.05	9.6	0.07	<.001
Health	12.1	0.05	12.0	0.09	.40	13.6	0.06	10.5	0.08	<.001
Physical activity	12.2	0.05	11.4	0.08	<.001	12.7	0.06	10.9	0.07	<.001
Dietary intake	22.6	0.08	22.5	0.15	.50	24.5	0.10	20.5	0.13	<.001
Completion time deemed acceptable for a NutriNet-Santé questionnaire	33.9	0.44	31.6	0.77	.004	36.4	0.51	29.2	0.68	<.001

^a^ Linear regression analyses mutually adjusted for gender, age, education, and level of computer skills. Mean completion time of questionnaires was log-transformed to improve normality.

**Table 6:** Comparison of mean response time for questionnaires (in minutes) according to educational level and self-evaluated computer skills, NutriNet-Santé Study, France, 2013.^a^

Type of questionnaire	Educational level					Level of computer skills				
	>12 y		≤12 y		*P*	Experienced		Novice		*P*
	Mean	SEM	Mean	SEM		Mean	SEM	Mean	SEM	
Sociodemographic	12.9	0.07	14.4	0.08	<.001	12.7	0.05	14.6	0.09	<.001
Anthropometric	9.6	0.06	11.6	0.07	<.001	9.9	0.05	11.3	0.08	<.001
Health	10.9	0.07	13.2	0.08	<.001	11.2	0.05	13.0	0.09	<.001
Physical activity	11.2	0.06	12.4	0.07	<.001	11.0	0.05	12.6	0.08	<.001
Dietary intake	22.5	0.11	22.5	0.12	.90	21.0	0.09	24.0	0.15	<.001
Completion time deemed acceptable for a NutriNet-Santé questionnaire	29.3	0.55	36.3	0.64	<.001	29.7	0.44	35.9	0.77	<.001

^a^ Linear regression analyses mutually adjusted for gender, age, education, and level of computer skills. Mean completion time of questionnaires was log-transformed to improve normality.

## Discussion

To our knowledge, this study is the first to shed light on computer skills and attitudes toward study demands of a large sample of French volunteers in a Web-based cohort. The main results showed that a substantial proportion of the participants (approximately one-quarter) declared being inexperienced or novice in computer use. Women tended to be more positive than men toward the study and its format, whereas participants with higher educational levels were less likely to be satisfied with the study demands, notably regarding the frequency and completion time of the questionnaires.

A key question in e-epidemiology pertains to the extent to which low computer skills represents a barrier to participation. Every year, the European Union Commission collects data on the digital skills of the population, measured by asking individuals if they had ever performed certain computer and/or Internet-related activities (Eurostat). In their 2012 report, 41% of the French population reported having either low or no computer skills [23]. The proportion of novice/inexperienced computer users was lower in our study population (24%). This was expected because access to the Internet was an inclusion criterion and since higher socio-professional categories were slightly (and commonly) overrepresented. Individuals in the latter category may have acquired a more practical and administrative use of the Web [24] given its regular use in the framework of their professional activity or during their university studies. Likewise, the proportion of NutriNet-Santé participants who could “install new devices,” “compress/decompress files,” or “use basic arithmetic formulas in a spreadsheet” was higher than observed in the French general population [25,26]. Our study population seemed to be better qualified for a more practical/working use of the Internet than for a leisure/entertainment use, with higher proportions of participants who could “send emails with attached files” or “fill in administrative forms online” and lower proportions of participants who could “download movies, music, games” or “upload texts, games, photos, movies, or music.” The older age of our cohort compared to the French general population probably contributes to explain these differences. However, a notable finding was that the proportion of computer novice/inexperienced participants was non-negligible suggesting that participation in Web-based cohorts is not restricted to computer experts. Given the rapid increase in digital skills among EU citizens [23], there is a marked trend of ever decreasing barriers related to computer literacy.

Interestingly, several categories of participants with lower computer skills (eg, women or participants with lower educational levels) were more positive toward the study and more accepting of the respondent burden. Indeed, overall satisfaction was high regarding the design of the study, with the majority of participants reporting support for parameters that are currently in effect in terms of questionnaire frequency (about 1/month) and time needed for completion (less than 30 minutes/questionnaire). However, sociodemographic characteristics modulated these opinions. In addition to their higher participation (76%, which was expected for a study related to nutrition questions), females tended to be more motivated and satisfied by the study than were their male counterparts. Unlike older adults (>50 years), younger adults felt more comfortable with the website and the questionnaires. Interestingly, participants with higher levels of education were somewhat less satisfied with the demands of study participation. This could be explained by several reasons. First, participants with higher educational levels are usually exposed to a variety of digital activities and Internet websites during their professional activities and their leisure time [24] and, thus, may be more demanding on the design and the usability of questionnaires. Second, as they spend more time on the Internet [27], they are more often deluged with all types of questionnaires via spam, making them less receptive to the questionnaires of the study. These results provide useful information for Web-based study protocol optimization. For instance, when applicable, ancillary protocols that are optional and do not necessarily necessitate the recruitment of representative subsamples of the cohort should only be geared toward the most receptive categories of participants identified in this study.

The proportion of respondents who preferred the current communication modes (email and Internet website compared to telephone, postal mail, or face-to-face interactions) was high (87%), especially in older participants, better educated and with higher computer skills. However, unlike some previous studies [28-30], this one was not designed as an experiment to test response rates according to survey mode, but only to evaluate the overall satisfaction level of participants regarding the survey mode. Thus, this figure should be toned down by the fact prior research indicates that respondents tend to prefer the mode they were interviewed in [31]. Overall, the proportion of participants interested in the development of a NutriNet-Santé smartphone app was relatively limited (21%). In fact, smartphone use in our study (34% in 2013, data not shown) was less widespread than in the general French population (50%) [32]. In our sample, interest in a smartphone app was strongly modulated by several parameters; notably, men and younger adults were those who demonstrated the highest interest. Thus, given the opportunity to participate in a research study via a smartphone application would provide a strategic opportunity to recruit participants who are currently underrepresented in the cohort.

Strengths of this study pertain to the use of a large population-based cohort sample and availability of detailed information on computer/Internet skills and attitude toward demands of an Internet-based study, in the context of growing interest in e-epidemiology coupled with scarce knowledge about these parameters.

Several limitations should be acknowledged. First, caution is needed in extrapolating our results to all Web-based investigations because the NutriNet-Santé study involved a sample of volunteers who accepted to participate in a survey on nutrition and health. Compared to national estimates [33], the NutriNet-Santé study included more women, older participants, and individuals belonging to higher socio-professional categories. Second, response rate to this specific nonmandatory computer/Internet skills questionnaire was 35%. In fact, nonresponse to this questionnaire did not alter the enrollment status of the participants. Compared to nonresponders, responders were more likely to be men, younger, and better educated. Thus, we may have underestimated the proportions of novice/inexperienced computer users. In addition, the questionnaire was administered 4 years after the launch of the cohort. Thus, it is possible that this survey underrepresented participants who might have dropped out of the study due to difficulties related to computer/Internet use. As is usually the case in prospective cohorts in which mandatory and nonmandatory questionnaires are sent to participants, the level of involvement varies between participants. When the computer literacy questionnaire was administered in October 2013, 78,380 participants were regular respondents to optional questionnaires (at least 1 questionnaire filled in over the last 6 months). Based on this population, response rate to the present questionnaire was higher (55%). Lastly, a social desirability bias may have occurred since computer and Internet skills were self-evaluated, which may have led to an overestimation of expertise. However, our method was similar to the one used by the EU Commission for such assessment [23].

In conclusion, this study provided new information on computer skills and attitude toward study demands according to sociodemographic profiles of participants involved in a large population-based Web cohort. These results are useful for optimizing current and future Web-based investigations, in the context of rapid development of e-epidemiology and the currently scarce e-methodology literature. A substantial part of the study population reported low computer/Internet skills, suggesting that this characteristic does not constitute a barrier to participation in Web-based cohorts. The finding further suggested that several categories of participants with lower computer skills (eg, women or participants with lower educational levels) were more positive toward the study and less reluctant to comply with its demands. This study also highlighted that developing a dedicated smartphone app may boost interest in participation among categories of participants who are relatively less represented in health-related e-cohorts, such as men and young adults.

## Abbreviations

CNAM: Conservatoire National des Arts et Métiers
FRM: Fondation pour la Recherche Médicale
INPES: Institut National de la Prévention et de l’Education pour la Santé
INRA: Institut National de la Recherche Agronomique
INSERM: Institut National de la Santé et de la Recherche Médicale
InVS: Institut de Veille Sanitaire
SEM: standard error of the mean
